# Optimizing Salvage ART: Real-World Outcomes of Doravirine Plus DTG or BIC in Heavily Treatment-Experienced Persons Living with HIV †

**DOI:** 10.3390/microorganisms14020390

**Published:** 2026-02-06

**Authors:** Irina Ianache, Roxana Radoi, Gratiela Tardei, Mike Youle, Cristiana Oprea

**Affiliations:** 1 Department of Infectious Diseases, Faculty of General Medicine, Carol Davila University for Medicine and Pharmacy, 020021 Bucharest, Romania; irina.ianache@umfcd.ro; 2 “Victor Babes” Clinical Hospital for Infectious and Tropical Diseases, 030303 Bucharest, Romania; dr_roxana_radoi@yahoo.com (R.R.); gtardei@spitalulbabes.ro (G.T.); 3 Royal Free Hospital, London NW3 2QG, UK; mike@mikeyoule.com

**Keywords:** PWH, salvage ART, treatment switch, heavily treatment-experienced PWH

## Abstract

Management of heavily treatment-experienced people living with HIV (HTE-PWH) remains challenging due to long antiretroviral therapy (ART) exposure and limited treatment options. We conducted an observational, real-world-data study on HTE-PWH in active care at the “Victor Babeș” Hospital, Bucharest, receiving doravirine (DOR)-based salvage regimens combined with dolutegravir (DTG) or bictegravir (BIC). Epidemiological, clinical, and laboratory variables were analyzed according to HIV acquisition mode and salvage regimen. Sixty-nine PWH were included; 57.9% male, with a median age of 36 years. Median ART duration before switch was 21.5 years. HIV was acquired parenterally in childhood (PM) in 64.7% cases. Salvage regimens included BIC/FTC/TAF + DOR (50.0%), 3TC/TDF/DOR + DTG (35.2%), and 3TC/DTG + DOR (14.7%). The median nadir CD4 count was 37 cells/µL, and the median viral load at diagnosis was 5.24 log10 copies/mL. Switching was performed for regimen simplification (n = 32) or non-adherence-related virological failure (n = 37). At switch, 53.6% had detectable viremia. Viral suppression was achieved in 68.3% at 6 months and 75.0% at 12 months. Individuals with PM infection were younger and had longer ART exposure than those with heterosexual acquisition. DOR-based salvage regimens combined with DTG or BIC were effective in adherent HTE-PWH, particularly those with extensive ART histories.

## 1. Introduction

The natural history of HIV was significantly influenced by the availability of efficient antiretroviral treatment (ART) with viral suppression, immune reconstitution and improvement of the quality and duration of life [1]. Persons living with HIV (PWH) nowadays have access to novel and efficient antiretroviral treatments (ART) based on high-genetic-barrier drugs, with fewer drug–drug interactions and adverse reactions. Treatment for newly diagnosed individuals is now much less of a challenge, with novel formulations and two-drug and single-tablet regimens [2,3]. However, over time, PWH may experience drug–drug interactions due to polypharmacy and aging, as well as adverse reactions and toxicities, or develop resistance-associated mutations, reducing their therapeutic options. This may result in poor clinical outcomes with increased mortality rates, as well as potentially increasing HIV transmission [2,4].

Heavily treatment-experienced (HTE) is a term currently used for PWH who only have a maximum of two available fully active ARV classes. The prevalence of HTE-PWH varies between geographical regions, impacted by access to integrase inhibitor (INSTI)-based regimens. Achieving and maintaining viral suppression for HTE-PWH is vital, as it improves immune function and reduces clinical disease [1,5,6].

In Romania between the late 1980s and the early 1990s, more than 13,000 children, the ‘HIV pediatric cohort‘, were parenterally infected due to unsafe medical practices in state-run hospitals and orphanages with HIV F1 clade, linked phylogenetically to the Angolan F subtype [7,8]. A significant number of children in hospitals and orphanages were infected, of whom half survived, and in 2025, more than five thousand are still alive. These children who grew up with HIV used all classes of antiretroviral drugs, and due to toxicity, high pill burden, and long treatment duration, many experienced “treatment fatigue” or poor adherence to treatment. The survivors of this pediatric cohort are now young HTE adults living with HIV. However, in the last decade, HIV in Romania has been acquired through sexual contact, both heterosexual (HSX) and same-sex contact (MSM), as well as by injecting drug use (PWIDs). PWIDs living with HIV often have associated neuro-psychiatric disorders and interactions between ART and co-medications or the illegal drugs they use, and many now have similar rates of HTE as those of the pediatric cohort [9,10,11].

The aim of our study was to assess epidemiological aspects and outcomes in PWH under salvage ART regimens based on a combination of a non-nucleoside reverse transcriptase inhibitor—NNRTI (DOR)—and an integrase inhibitor—dolutegravir (DTG) or bictegravir (BIC)—in Romania, as few data on this subject have been published.

## 2. Materials and Methods

### 2.1. Patient Population

We conducted an observational, real-world-data study on PWH in active care between 1 January and 31 December 2023 at “Victor Babes” HIV Regional Center, Bucharest, Romania, with a prescribed regimen containing doravirine with either dolutegravir or bictegravir.

Variables of interest were related to socio-demographic and clinical characteristics (age, gender, ART history, comorbidities, reasons for switch), immuno-virological status (CD4 cell count, nadir CD4, plasma viral load at baseline and 6 and 12 months after the switch, respectively) and outcomes. The baseline HIV-RNA value was deemed the last evaluation before the switch. Reasons for switching to a salvage regimen included virologic failure, toxicities, or simplification of a complex ART regimen. These variables were collated from the electronic database and were compared based on HIV mode of acquisition—parenteral mode of HIV acquisition during childhood (PM) and non-PM (HSX, MSM and PWIDs) or salvage ARV regimen (BIC/FTC/TAF + DOR, 3TC/TDF/DOR + DTG and 3TC/DTG + DOR).

### 2.2. Laboratory Methods

HIV-1 viral loads were measured on Cobas^®^ 6800 instrument (Roche, Basel, Switzerland), with Cobas^®^ HIV-1 test, from Roche, Basel, Switzerland.

CD4 counts were measured using the BD FACS CANTO II flow-cytometer, with BD Multitest™ BD Multitest™ CD3 FITC/CD8 PE/CD45 PerCP/CD4 APC reagent, from Becton Dickinson Biosciences, San Jose, CA, USA.

RNA-HIV not detected (ND) was defined as a viral load (VL) < 40 copies/mL.

However, we reported a threshold of 50 copies/mL because this cut-off is commonly used in data presentation, ensuring consistency and comparability with published literature.

### 2.3. Statistical Analysis

Statistical comparisons were performed using SPSS vs 20.1. We summarized socio-demographic, clinical and immuno-virological characteristics using interquartile range (IQR) or absolute count (n) and percentage (%) as appropriate. Quantitative variables (CD4 cell count, HIV-RNA value, age, duration on ART) were compared using Mann–Whitney test and qualitative ones (gender, modes of HIV acquisition) using Chi-square test. Statistically significant difference was considered in cases of *p*-values of less than 0.05.

## 3. Results

### 3.1. Socio-Demographic and Clinical Characteristics of PWH on Salvage ART Regimen

During the twelve months of the study, a total of 2710 PWH were in active care, out of which sixty-nine were heavily exposed to ART and were switched or changed to ART salvage regimens. More than half of them, 40 (57.9%), were males, with a median age of 36 years (IQR 35–49). Modes of HIV acquisition were parenteral mode during childhood (PM) 44 (64.7%), heterosexual contact (HSX) 18 (6.4%), injecting drug use (PWID) 3 (4.3%), men having sex with men (MSM) 1 (1.4%) and vertical transmission 3 (4.3%) (Table 1).

Forty-eight (70.5%) PWH had at least one comorbidity, with dyslipidemia (28), neuro-psychiatric (12), cardiovascular (10), diabetes (6) and neoplasia (5) being the most frequent.

ART was switched to a salvage regimen after a median time of 21.5 years (IQR 15.75–24). The median number of previous ART regimens was seven (IQR 5–8.25), with a median time of 15 (IQR 8–20) years under protease inhibitors (PIs), 6 (IQR 3–10) under INSTI and 4 (IQR 2–10) under non-nucleoside reverse transcriptase inhibitors (NNRTIs). The median nadir CD4/µL and CD4/μL at change were 37 (IQR 15–124) and 296 (IQR 151–640), respectively, and the median HIV viral load (log_10_copies/mL) at HIV diagnosis was 5.24 (IQR 4.71–5.69). Only 31 (45.5%) PWH were undetectable at switch (Table 2).

### 3.2. Salvage ART Regimens

The reasons for switching were virological failure in 37 (53.6%) and simplification in 32 (46.3%) subjects. ART salvage regimens used were BIC/FTC/TAF + DOR (35, 50.7%), 3TC/TDF/DOR + DTG (24, 35.2%) and 3TC/DTG + DOR (10, 14.7%). Immunological, virological and ART history characteristics were similar irrespective of ART salvage regimen, with no statistically significant differences (Table 3). However, 3TC/DTG + DOR had a higher CD4 cell count (*p* = 0.049), being used more frequently in PWH adherent to treatment but with toxicities to nucleoside reverse transcriptase inhibitors (NRTIs).

We also compared socio-demographic and clinical characteristics based on HIV mode of acquisition. Compared to individuals with a heterosexual mode of HIV acquisition, those infected parenterally during childhood were younger (*p* = 0.01) and had a longer duration on ART. However, when we compared PM with all other groups (non-PM), there was no statistically significant difference related to age, but the median duration on ART (all classes) before the initiation of the salvage regimen was still higher in the PM group (Table 4).

### 3.3. Virological Suppression Rates Under Salvage ARV Regimens

**Figure 1:**
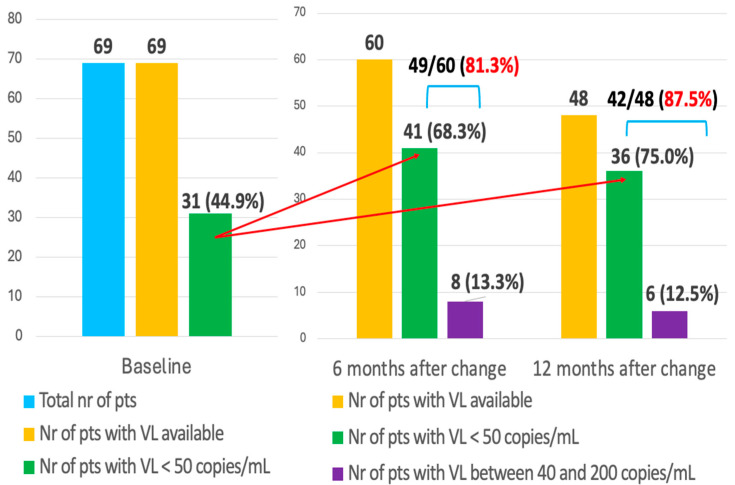
**Evaluation of HIV-RNA in plasma after switching to salvage ART regimen.**

We evaluated the viral suppression rate after the switch to salvage regimens in patients with undetectable HIV VL at baseline. After 6 months, we registered a significant increase in the number of individuals with undetectable HIV viral load (67.7%), and 81.3% had a viral load below 200 copies/mL. These rates were even higher 12 months after the switch, when 76.5% of subjects with available viremia were undetectable, and almost 90% had VL < 200 copies/mL (Figure 1).

**Figure 2:**
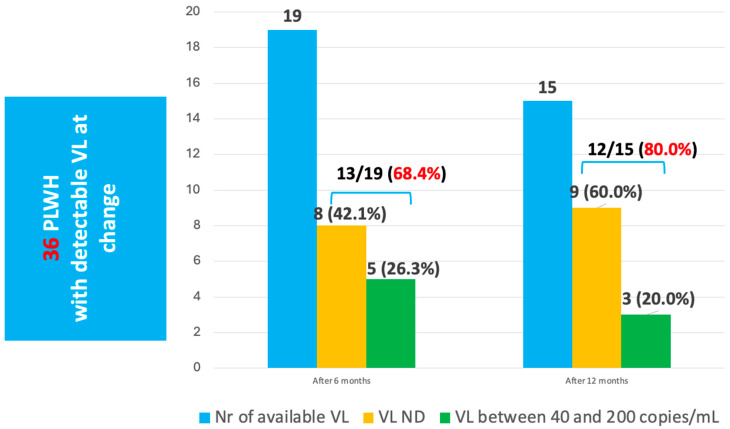
**Changes in plasma HIV RNA after initiation of salvage ART regimens in PWH with detectable VL at baseline.**

We also followed up with the 36 patients with detectable viral loads at baseline. After 6 months the HIV viral load was available for nineteen people, out of which eight (42.1%) were undetectable and 13 (68.4%) had a VL below 200 copies/mL. Similarly, the rates of viral suppression increased at 12 months after the change, with 60.0% being undetectable and 80.0% with low-level viremia (Figure 2).

## 4. Discussion

In this study we evaluated the outcomes of doravirine plus second-generation integrase inhibitor-based therapies as salvage regimens in HTE-PWH in active care at a Romanian tertiary healthcare facility, comparing outcomes by mode of acquisition.

As stated, Romania has a unique cohort of PWH infected parenterally during childhood with 5000 long-term survivors, now being young adults between 34 and 36 years of age [8,10,11,12]. These PWH received ART since childhood, when the first antiretrovirals became available in Romania, including monotherapy or dual therapy, with ART that are no longer available, having been withdrawn due to toxicities. However, more than half of PWH from this cohort are still alive due to significant efforts made by healthcare professionals to provide access to treatment. Moreover, during their childhood and as young adults, they experienced a lot of stigma and discrimination, and due to the use of toxic drugs, most of them developed lipodystrophy [10,11,12,13].

During the last decades, HIV acquisition in Romania has mainly been sexual, with both different- and same-sex partners, heterosexual transmission of HIV being nowadays the most prevalent one. A high proportion of persons newly diagnosed with HIV are MSMs, who are often young, educated males adherent to treatment, who rarely need salvage ART regimen. Injecting drug use is still a challenge for HIV control in Romania, but the proportion of newly diagnosed cases among injecting drug users significantly decreased since new psychoactive drugs (“ethnobotanical drugs”) became illegal in 2014 [7,10,11,14,15].

Previous studies have shown that PWH with limited treatment options are often older individuals with low CD4 cell counts and high HIV viral load, with a history of NRTI-based mono or dual therapy [5,16]. These characteristics describe the profile of cases from our cohort, except for their age.

Heavily treatment-experienced PWH have limited ART options, and the initial definition of the number of previously used ART regimens was updated, since the actual standard of care includes ART switches in virologically suppressed PWH for simplification to improved available therapies [1]. The patients enrolled in the study were HTE-PWH, irrespective of definitions, with a median of seven previous ART regimens, and almost half, irrespective of the mode of HIV acquisition, were undetectable at switch, which was chosen for the simplification of complex regimens or to avoid toxicities or drug–drug interactions. In the literature, we found very few reports about HTE-PWH being switched to salvage regimens for ART simplification or to reduce the risk of toxicities.

In this study protease inhibitors had the longest median duration of use, followed by integrase inhibitors and non-nucleoside reverse-transcriptase inhibitors. PI-associated mutations are rare in PWH whose first ARV regimen was based on PIs, being more often identified in HTE-PWH who initiated ART in the pre-ART era with nucleoside-based mono or dual therapies and later with unboosted first-generation PIs. These individuals often possess high-level resistance mutations to multiple PIs, including those with a high genetic barrier [17]. Subjects belonging to the HIV pediatric cohort had a significantly higher treatment duration on all classes of antiretrovirals, especially protease inhibitors. More than half of PWH from our study had taken all classes of antiretrovirals and regimens, including mono and dual therapies, with a high probability of significant resistance, explaining virological failure and the importance of salvage ARV regimens in this cohort.

When INSTIs became available in 2006, the longer duration compared to NNRTIs in our HTE-PWH may be explained by the immediate switch to regimens based on this new class of antiretrovirals in PWH with the failure of previous old regimens. In addition, during the last decade, guidelines recommended INSTI-based regimens as the first-line treatment, so all newly diagnosed PWH initiated treatment with these drugs [1].

Even if young by age, the PWH infected in childhood are old by disease. The chronic inflammation generated by HIV due to microbial translocation and damage-associated molecular patterns, with immune activation and proinflammatory cytokines, leads to premature aging and more comorbidities even in young adults. Unfortunately, ART cannot completely restore the T- and B-cell phenotypes, and many persons living with HIV from childhood have accelerated telomere attrition, as well as exhausted and senescent cells, with high risks of developing comorbidities that are more common in the older general population and require association of multiple medications with risk of drug–drug interactions and toxicities [18]. Dyslipidemia, cardiovascular diseases, diabetes, and neoplasms were the most common comorbidities in our cohort, reflecting long-term use of PIs in our cohort.

PWH treated with PI-based regimens, including newer ones, have an elevated risk of coronary heart disease due to alterations in plasma lipids and insulin levels. As prevention, these PWH require statin-based treatments that also lead to drug–drug interactions and adverse reactions, like myopathy or hepatotoxicity. We also identified a high number of neuro-psychiatric disorders, more often in subjects belonging to the pediatric cohort, including depression, anxiety, panic attacks and intellectual disability. Living with HIV since childhood and facing stigma was a challenge for these people, leading to poor adherence to treatment and even the risk of suicide. In order to prevent these events, psychotherapeutic and pharmacological interventions were required [19,20,21].

Novel agents have recently been approved for use in HTE-PWH (ibalizumab, fostemsavir and lenacapavir), all with novel mechanisms of action. Data available so far suggest that, when administered with efficient background regimens, each of these are able to achieve viral suppression in HTE-PWH [22]. Unfortunately, access to these novel medications is limited to some high-resource settings, and they are currently not available in Romania.

Access to genotyping testing in Romania can be limited, so changes to salvage regimens were, in most cases, made without analyzing the resistance-associated mutation profile. The salvage ART regimens used in our study, based on a combination of a non-nucleoside reverse transcriptase inhibitor, doravirine, and an integrase inhibitor—dolutegravir or bictegravir—were very effective in our cohort, as also suggested by other studies [16].

Our outcomes under salvage ART regimens were favorable, with more than 80% of HTE PWH achieving viral loads <200 copies/mL, with even higher rates after 12 months. PWH who were undetectable at ART change maintained their viral suppression, and more than half of those with virologic failure managed to reach viral suppression under the salvage ART regimen, demonstrating once again the efficacy of these combinations.

There remains uncertainty on the best option to simplify extensive ART regimens in HTE-PWH with good adherence and undetectable plasma HIV-RNA: whether an integrase inhibitor-based single tablet regimen (STR) could be sufficient, or if it would still be safer to use a salvage ART regimen.

The results of the “DoDo” study, which proposed an alternative antiretroviral 2-drug regimen of doravirine and dolutegravir for heavily pre-treated patients, showed that this double combination could be a valuable option for ART optimization in persons multiple experienced to treatment who have undetectable loads at switch [23].

There are few published studies describing the experience with ART switch on off-label dual regimens based on DOR and DTG; the majority suggest efficacy and viral replication control. Switching to this regimen was performed in most cases to avoid drug–drug interactions and toxicities; the conclusion obtained was that performing the switch in the absence of genotyping tests and data about resistance-associated mutations is risky, but our study suggests otherwise and that it and other novel combinations can be a suitable option in resource-limited settings [23,24,25].

### Limitations

One limitation was a lack of viral-resistance testing. Genotyping was not measured for all patients due to poor adherence to treatment or due to low-level viremia, which makes genotyping tests difficult. The results of the study were based on data from a single clinical center. Although a high proportion of PWH from the south-eastern region of Romania are seen in our center, more generalizable results would have emerged had we collected data from more areas of the country.

## 5. Conclusions

The number of PWH who switched to salvage ARV regimens in Romania significantly increased during recent years, especially among subjects belonging to the HIV pediatric cohort, with long durations of ART. The salvage ART regimens containing doravirine and dolutegravir or bictegravir all improved outcomes and increased the rates of undetectable HIV RNA in adherent PWH. More data is required to prove if STRs with high genetic barriers are an efficient and durable option in PWH with extensive treatment history or if double regimens with DTG and DOR could also be effective in this group. Our study showed that these salvage regimens, with high genetic barriers to resistance, were efficient even in the absence of prior resistance testing.

## Figures and Tables

**Table 1:** **Socio-demographic characteristics of PWH on salvage ARV regimens.**

Characteristics		Total n = 69
Male sex	n (%)	40 (57.9)
Age (years)	median (IQR)	36 (35–49)
Mode of HIV acquisition		
parenteral mode during childhood (PM)	median (IQR)	44 (64.7)
heterosexual contact (HSX)		18 (26.4)
injecting drug use (PWID)		3 (4.3)
men having sex with men (MSM)		1 (1.4)
vertical transmission		3 (4.3)

Legend: PM = parenteral mode of HIV acquisition during childhood; HSX = heterosexual contact; PWID = people who inject drugs; MSM = men who have sex with men.

**Table 2:** **ART history and immuno-virological aspects in PWH on salvage ART regimens.**

Characteristics		Total n = 69
Duration on ART before change (years)	median (IQR)	21.5 (15.75–24)
Nadir CD4 cell count/μL	median (IQR)	37 (15–124)
HIV-RNA (log10copies/mL) at diagnosis	median (IQR)	5.24 (4.71–5.69)
CD4 cell count/μL at change	median (IQR)	296 (151–640)
HIV-RNA at change—ND	n (%)	31 (45.5)
No. of previous ART regimens	median (IQR)	7 (5–8.25)
Duration under INSTI (years)	median (IQR)	6 (3–10)
Duration under PIs (years)	median (IQR)	15 (8–20)
Duration under NNRTIs (years)	median (IQR)	4 (2–10)

Legend: ART = antiretroviral treatment; ND = not detected; INSTIs = integrase inhibitors; PIs = protease inhibitors; NNRTIs = non-nucleoside reverse transcriptase inhibitors.

**Table 3:** **Characteristics of PWH on salvage ART regimens—comparison based on ART regimen.**

Characteristics		BIC/FTC/TAF + DOR N = 35	3TC/TDF/DOR + DTG N = 24	3TC/DTG + DOR N = 10	*p* Value
Nadir CD4 cell count/μL	median (IQR)	75 (21–135)	44.5 (10–115.5)	73.5 (22.7–115.5)	0.610
HIV-RNA at diagnosis log_10_copies/mL	median (IQR)	5.08 (4.08–5.69)	5.16 (4.81–5.44)	5.76 (5.45–5.96)	0.187
CD4 cell count at change/μL	median (IQR)	305 (163–637)	220 (101–452.5)	755 (381.5–1035.5)	0.049
HIV-RNA at change—ND	n (%)	16 (47.0)	8 (33.3)	7 (70.0)	0.143
Prior ART regimens	n (%)	7 (5–8.75)	7 (4.5–8)	5.5 (5–8.5)	0.874
Duration under INSTI (years)	median (IQR)	4 (2–10)	6 (4.25–7.75)	8 (5–14)	0.096
Duration of PIs (years)	median (IQR)	16.5 (10–21.25)	13.5 (7.75–17.75)	12 (5.5–17.5)	0.339
Duration of NNRTIs (years)	median (IQR)	4 (2–10.5)	3 (1–9)	6.5 (3–11)	0.529

Legend: ND = not detected; INSTIs = integrase inhibitors; PIs = protease inhibitors; NNRTIs = non-nucleoside reverse transcriptase inhibitors.

**Table 4:** **Characteristics of PWH on salvage ART regimens—comparison based on modes of HIV acquisition.**

Characteristics		PM N = 44	Non-PM N = 25	*p* Value
Male sex	n (%)	24 (54.5)	15 (60.0)	0.612
Age (years)	median (IQR)	35.5 (35–36)	44 (34–54.2)	0.125
Duration of ART before change	median (IQR)	23.5 (21–25)	11 (6.75–16.5)	<0.0001
Nadir CD4 cell count/μL	median (IQR)	80 (12.5–113)	49.5 (16.7–144.5)	0.814
HIV-RNA (log_10_copies/mL) at diagnosis	median (IQR)	5.23 (4.90–5.69)	5.26 (4.55–5.69)	0.957
CD4 cell count at ART change/μL	median (IQR)	309.5 (175–684.25)	230.5 (86.25–509.5)	0.142
HIV-RNA at change—ND	n (%)	21 (47.7)	9 (36.0)	0.472
Prior ART regimens	n (%)	7 (5.75–9)	5 (4–6)	<0.0001
Duration on INSTI (years)	median (IQR)	7 (4–12)	5 (3.5–6)	<0.0001
Duration of PIs (years)	median (IQR)	17 (10–21.5)	4 (2.5–6)	0.009
Duration on NNRTIs (years)	median (IQR)	6 (3–10)	6 (2.75–15)	<0.0001

**Legend:** ND = not detected; INSTIs = integrase inhibitors; PIs = protease inhibitors; NNRTIs = non-nucleoside reverse transcriptase inhibitors.

## Data Availability

The original contributions presented in this study are included in the article. Further inquiries can be directed to the corresponding author. The raw patient data are preserved in the electronic database of Victor Babes Clinical Hospital for Infectious and Tropical Diseases, Bucharest, Romania, and consist of their files (diagnosis, history, outcome, and all laboratory tests).

## Abbreviations

The following abbreviations are used in this manuscript:

ART	Antiretroviral treatment
PWH	Persons living with HIV
THE	Heavily treatment-experienced
INSTIs	Integrase inhibitors
HIV	Human immunodeficiency virus
HSX	Heterosexuals
PWIDs	Persons who inject drugs
DOR	Doravirine
DTG	Dolutegravir
BIC	Bictegravir
3TC	Lamivudine
FTC	Emtricitabine
TDF	Tenofovir disoproxil fumarate
TAF	Tenofovir alafenamide fumarate
MSM	Men having sex with men
ND	Not detectable
VL	Viral load
PM	Parenteral mode of HIV acquisition during childhood
PIs	Protease inhibitors
NNRTIs	Non-nucleoside reverse transcriptase inhibitors
NRTIs	Nucleoside reverse transcriptase inhibitors
STR	Single-tablet regimen
